# Transcriptomic Analysis of Ovine Hepatic Lymph Node Following *Fasciola hepatica* Infection – Inhibition of NK Cell and IgE-Mediated Signaling

**DOI:** 10.3389/fimmu.2021.687579

**Published:** 2021-05-28

**Authors:** Amalia Naranjo-Lucena, Carolina N. Correia, Verónica Molina-Hernández, Álvaro Martínez-Moreno, John A. Browne, José Pérez, David E. MacHugh, Grace Mulcahy

**Affiliations:** ^1^ UCD School of Veterinary Medicine, Dublin, Ireland; ^2^ Animal Genomics Laboratory, UCD School of Agriculture and Food Science, Dublin, Ireland; ^3^ Departamento de Anatomía y Anatomía Patológica Comparadas y Toxicología, Facultad de Veterinaria, Universidad de Córdoba, Córdoba, Spain; ^4^ Departamento de Sanidad Animal (Parasitología), Facultad de Veterinaria, Universidad de Córdoba, Córdoba, Spain; ^5^ UCD Conway Institute of Biomolecular and Biomedical Research, Dublin, Ireland

**Keywords:** transcriptomics, sheep, lymph node, NK cells, *Fasciola*, IgE signalling

## Abstract

*Fasciola hepatica* is a trematode parasite responsible for major economic losses in livestock production, and is also a food-borne zoonotic agent in developing rural regions. For years, the immunoregulatory mechanisms employed by the parasite have hampered efforts to develop a successful vaccine candidate. Given that a comprehensive understanding of the immune response to infection is needed, we investigated the gene expression changes in ovine hepatic lymph nodes after experimental infection with *F. hepatica*. Lymph nodes from uninfected and infected animals were processed for RNA sequencing (RNA-seq) at 16 weeks post-infection. Comparison of groups revealed 5,132 differentially-expressed genes (DEGs). An inhibition of pro-inflammatory pathways, which has previously been described during fasciolosis, was evident in our data. However, other signals previously identified in ruminant peripheral blood mononuclear cells (PBMC) or liver tissue, such as activation of TGF-β or apoptosis-related pathways were not detected. We found inhibition of some key immunological pathways, including natural killer (NK) cell activity and IgE-mediated signaling. These may point to additional some as yet unrecognized mechanisms employed by the parasite to evade the host immune response. Understanding these, and leveraging information from this and other omics studies, will be important for the development of future vaccine prototypes against this parasite.

## Introduction

*Fasciola hepatica*, the liver fluke, is a parasite of livestock, wildlife and humans, present worldwide, except for Antarctica. It is a very significant cause of economic losses to animal agriculture, and the options for control of infection are limited, with resistance against flukicides among fluke populations increasing (1, 2). Development of a vaccine, even if only partially protective, would contribute to the sustainability of global agriculture and animal welfare by reducing costs of animal production and decreasing the use of anthelmintics (3).

Sheep can experience acute (sometimes fatal), subacute or chronic disease once they become infected, while chronic infections are most common in cattle (4, 5). The reasons for this difference in disease presentation are however still unknown. It has been suggested that cattle have a denser hepatic parenchyma, which might slow down the invasion by the flukes and reduce pathology. On the other hand, differences in the immune response to infection may account for differences in host susceptibility. In the initial stages of infection there is a mixed systemic response with activation of T_h_1/T_h_2/Treg pathways, while in chronic infection the immune response shifts towards T_h_2 activation, with sheep usually showing this pattern earlier than cattle (6–8). It has been shown that this shift is at least partly induced by molecules released by the parasite. Some effects observed include alternative activation of macrophages, impairment of mast cells and the ability of dendritic cells to drive T_h_1 responses or reduction of lymphocyte responsiveness, among others (8–11). These immunomodulatory abilities presumably allow for long-term survival of the parasite within the host.

Considerable efforts to develop a vaccine that can at least partially protect ruminants have been made (3). However, the immunomodulating mechanisms employed by *F. hepatica* have hampered these efforts. Therefore, a more in-depth knowledge of the immune response is necessary to expedite the investigation of new vaccine candidates and vaccination strategies. Previous studies have used transcriptomics to analyze the systemic immune response in cattle and sheep (6, 7, 12), and the local response in the liver tissue of sheep (13). However, to the best of our knowledge, this is the first transcriptomic analysis investigating the immunological changes produced by infection with *F. hepatica* within the hepatic lymph nodes of sheep (*Ovis aries*). This study helps us to further understand the mechanisms employed by *F. hepatica* to subvert the host immune response at a local level.

## Methods

### Ethics and Experimental Design

This experiment was approved by the Bioethics Committee of the University of Córdoba (UCO, Spain) (code No. 1118) and conducted in accordance with European (2010/63/UE) and Spanish (RD 1201/2005) Directives on animal experimentation. The animals formed the control groups of a separate vaccine trial (not reported here), in which five groups of animals were included: infected positive control, uninfected negative control, vaccine 1, vaccine 2 and vaccine 3. For the purpose of our study, only the positive and negative control groups were analyzed. We will refer only to those groups hereafter.

Nineteen male Merino-breed sheep, between six and nine-months of age, sourced from a liver fluke-free farm were included. Animals were housed at the Research Farm of the University of Córdoba (100 m^2^ covered and 100 m^2^ uncovered facilities) throughout the trial and were fed hay and pellets and provided with water *ad libitum*. Prior to inclusion, all animals were tested for fluke eggs by faecal sedimentation, with no eggs detected (14). Additionally, all animals were tested nine weeks before infection for serum IgG specific for *F. hepatica* cathepsin L1 (FhCL1) using an enzyme-linked immunosorbent assay (ELISA) as previously described by Clery et al. (8), with negative results in all cases (8). Sheep were randomly allocated to Group 1 (*n* = 11) infected, and Group 2 (*n* = 8) uninfected. The number of allocated sheep in each group was decided based on Hart et al. (15), with a minimum of 8 animals per group (15). Sheep from Group 1 were orally infected with a single dose of 120 *F. hepatica* metacercariae of the Ridgeway (Gloucestershire, UK) strain, and all animals were kept in the same conditions for 16 weeks thereafter.

### Sampling Procedure and Slaughter

Blood samples were collected in 10 ml EDTA vacutainers (BD) by jugular venipuncture at the day of infection (pre-infection), 2 weeks post-infection (wpi), 7 wpi and 16 wpi. Animals were weighed at the beginning and at the end of the trial. Total and differential haematological data was generated using a ProCyte Dx Hematology Analyzer, and included information on eosinophils, lymphocytes, monocytes, neutrophils, basophils, PVC and haemoglobin. Total plasma proteins were determined using a manual refractometer. Faecal samples were obtained at 7, 8, 9, 10, 11, 12, 13 and 15 wpi to quantify fluke faecal egg counts (FEC) by sedimentation. No adverse reactions or clinical signs were noted throughout the trial. All animals were euthanized in batches of five at 16 weeks post-infection by intravenous injection of T61^®^ (MSD Animal Health, Salamanca, Spain) according to the manufacturer’s instructions. Post-mortem examination was performed at the facilities of UCO. Liver and hepatic lymph node from all animals were obtained and weighed. Gross liver pathology was scored as mild (0-10% liver surface affected), moderate (10-20%) severe (20-30%) and very severe (+30%) and fluke burden for each animal was estimated as previously described (16). Sections of hepatic lymph nodes comprising relevant histological structures (cortex and medulla) were obtained and placed in cryotubes to be snap frozen in liquid nitrogen. Tissue samples were kept in liquid nitrogen until used. Sections of the hepatic lymph nodes were fixed in 10% buffered formalin and embedded in paraffin wax for histopathology analysis. Tissue sections (4 μm) were stained with haematoxylin and eosin for histopathology examination. Gross and histopathology were examined blindly by two pathologists. Hyperplasia of lymphoid follicles, interfollicular areas and medullary cords from infected animals were scored with respect to uninfected controls.

When statistical comparison was performed between infected and control groups, data was checked for normality using the D’Agostino-Pearson test and the Unpaired t-test or Mann-Whitney test were used for group analyses of normally and not normally distributed data respectively.

### RNA Extraction From Hepatic Lymph Nodes

Total hepatic lymph node RNA from the 11 infected (16 wpi) and 8 uninfected animals was isolated by homogenising approximately 50 mg of tissue in 1.0 ml of TRIzol reagent (Invitrogen). The RNA was further purified by a column clean-up using the Qiagen RNeasy mini kit (Qiagen) as per the manufacturer’s instructions. RNA quantification was performed using a Nanodrop (One/One^C^ Microvolume UV-Vis) spectrophotometer (Thermo Fisher Scientific) while RNA quality was evaluated using an Agilent 2100 Bioanalyzer with an RNA 6000 Nano Lab Chip Kit (Agilent Technologies). All samples had A260/280 ratio >2.0 and RNA integrity number (RIN) >7.3.

### Library Preparation and Sequencing

Five micrograms of total RNA per sample were sent to Brigham Young University DNA Sequencing Center (BYU DNASC) for library preparation and sequencing. Barcoded paired-end (PE) libraries were prepared using a KAPA stranded mRNA-seq kit and KAPA Dual-Indexed Adapter Kit (Roche), following the manufacturer’s instructions. Quality control was performed and a pool of all 19 barcoded samples was loaded onto an Illumina NovaSeq 6000 Sequencing System flow cell at 2 × 150 nucleotide reads.

### Differential Expression Analysis

﻿Bioinformatics analyses were performed using scripts developed in GNU bash version 4.4.19 (http://ftp.gnu.org/gnu/bash), and R version 4.0.0. All scripts can be accessed at a GitHub public repository (https://github.com/anaranjolucena/Ovine_LN_RNAseq). FASTQ files and corresponding MD5 values were stored on the BYU DNASC compute cluster and downloaded to a server at UCD. The integrity of the FASTQ files was checked using md5sum version 8.28. FASTQ files were then evaluated with FastQC v0.11.8 (www.bioinformatics.babraham.ac.uk/projects/fastqc). The ngsShoRT v2.2 software package (17) was used to filter out adapter sequences, remove poor quality reads (Phred Score (Q) lower than 20 for more than 25% of the nucleotides) and remove reads that were shorter than 151 nucleotides. FASTQ files were then re-assessed with FastQC. Filtered PE reads were aligned and annotated to the ovine reference genome (Oar_rambouillet_v1.0), which was downloaded from the National Center for Biotechnology Information (https://ftp.ncbi.nlm.nih.gov/genomes/refseq/vertebrate_mammalian/Ovis_aries/latest_assembly_versions), using alignment software STAR v2.7.3a (18). Mapping of reads against locations in the sheep genome was visualised using the Integrative Genomics Viewer v2.4.19 (19). Unmapped and multimapping reads were eliminated and the featureCounts program from the Subread v2.2.2 software package (20) was used to generate gene counts.

Gene annotation information was extracted from the GTF file for the reference genome. Genes with low expression levels of <1 count per million (cpm) in 7 or more libraries were discarded from further analysis. Normalization factors were then calculated using the trimmed mean of M-values (TMM) with the R/Bioconductor edgeR package v3.30.0 (21). Principal component analysis (PCA) was performed using scaled log_2_ cpm from filtered counts with the prcomp function and hierarchical clusters were assessed by calculating distances based on cpm and using the hclust function. Both functions are from the R stats package (R version 4.0.0 standard library). A design matrix was created to compare control and infected animals. Common and trended dispersions were estimated using the Cox-Reid profile-adjusted likelihood function (22) and tagwise dispersion was estimated with the empirical Bayes method to fit a quasi-likelihood negative binomial generalized linear model. Differentially expressed genes (DEGs) were determined by a quasi-likelihood *F*-test (23) using a false discovery rate (FDR) cut-off of 5%.

### Ingenuity Pathway Analysis (IPA)

Qiagen Ingenuity Pathway Analysis (﻿IPA; https://digitalinsights.qiagen.com/qiagen-ipa) does not support the upload of sheep gene identifiers; therefore, sheep-to-human ortholog genes were retrieved using OrthoDB (https://www.orthodb.org) (24). Only human genes with a one-to-one pairwise orthology relationship to ovine genes were used in the IPA analysis. Canonical pathways were considered significantly enriched if they exhibited a *P*-value < 0.05, and |Z-score values| >2 were used to predict the activation or inhibition status of upstream and master regulators, or increased or decreased representation of a disease or function (25).

### Gene Ontology (GO) and Kyoto Encyclopaedia of Genes and Genomes (KEGG)

The Database for Annotation, Visualization and Integrated Discovery (DAVID), Bioinformatics Resources version 6.8 (26) (https://david.ncifcrf.gov) was used to obtain significantly enriched Gene Ontology (GO) Biological Process terms and Kyoto Encyclopaedia of Genes and Genomes (KEGG) pathways. GO terms and KEGG pathways with *P-*value < 0.05 were considered to be significantly enriched.

## Results

### Experimental Infection

**Figure 1:**
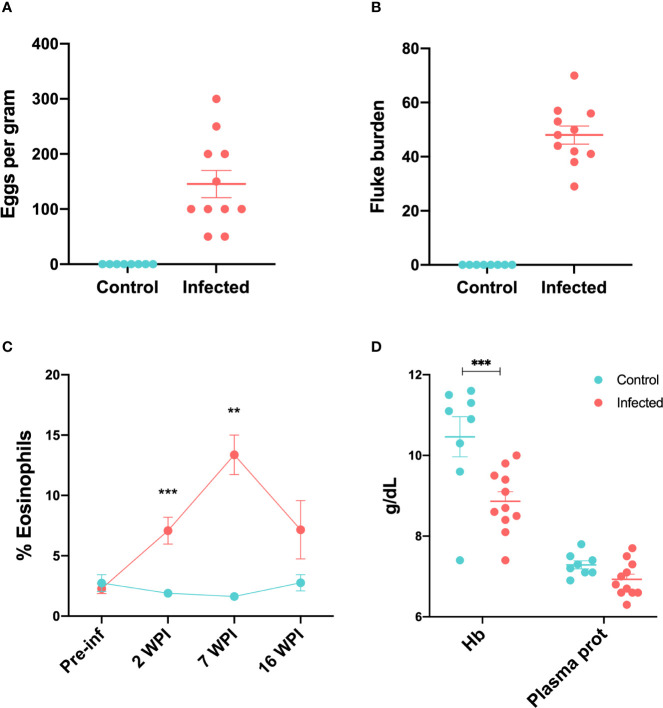
Parasitological, haematological and serum biochemical changes in infected and control sheep **(A)** Faecal egg counts showed as eggs per gram at 15 wpi **(B)** Fluke burdens obtained at post-mortem 15 wpi. **(C)** Percentage of peripheral eosinophils in control and infected animals throughout the infection trial. Data from all 11 infected and 8 control animals is included in each time-point except for 7 wpi, where data from 7 infected and 4 control animals were available. **(D)** Concentration of haemoglobin and total plasma proteins at 16 wpi in control and infected animals. Bars represent mean and standard error of the mean. **P *<* 0.01, ***P *<* 0.001.

Prior to experimental infection, all animals were clinically normal. Average weight was 48.37 kg (SD=10.10). By week nine after infection, two animals from the infected group started to show the presence of *F. hepatica* eggs in their faeces, and eggs were detected in all 11 infected animals by the end of the study, with fluke eggs per gram varying between 50 and 450 (**Figure 1A**). No eggs were detected in the control animals. At post-mortem examination, no flukes were detected in control animals. **Figure 1B** shows the fluke burden observed at post-mortem examination in infected animals.

Infected animals showed an increased percentage of peripheral eosinophils compared to the control group at week 2 after infection (*P* < 0.01). This peaked by week 7 (*P* < 0.01) (**Figure 1C**). There were no significant differences between control and infected groups with respect to peripheral lymphocytes, monocytes, neutrophils, basophils or packed cell volume (PCV) throughout the trial (**Supplementary Figure 1**). Total plasma protein in infected animals at 16 wpi was lower than in control animals, although this difference was not significant. Haemoglobin concentrations were significantly lower in the infected group (*P* < 0.001*)* at 16 wpi (**Figure 1D**).

The average weight gain by the end of the trial was 1.03 kg (SD= 2.19) for the infected animals and 2.1 (SD=2.41) for the uninfected group. The weight of livers at post-mortem was 526.97 g (SD=94.60) for uninfected animals and 670.67 g (SD=74.54) for infected animals, which was significantly different by unpaired t-test (*P* *=* 0.017). Mean score for gross hepatic lesions in the infected group was 3.0 (SD=0.89) range 2-4.

Hepatic lymph nodes were also enlarged in infected animals, with an average weight of 8.45 g (SD=6.44) as compared with those from uninfected animals at 1.52 g (SD=1.03). These differences were significant by the Mann Whitney test (*P* *<* 0.0001). The most relevant histological changes of hepatic lymph nodes from the infected group versus the uninfected group were marked hyperplasia of lymphoid follicles with large germinal centres, which resulted in an average histopathological score of 3.73 (SD=0.47). Interfollicular or paracortical areas and medullary cords were also hyperplastic with abundant mature lymphocytes resulting in a score of 2.82 (SD=0.60) and 2.73 (SD=0.47), respectively. Some hepatic lymph nodes from infected animals showed moderate infiltration of eosinophils while none of them showed fibrosis (**Supplementary Figure 2**). Data for haematology, pathology and parasitology results can be found in **Supplementary Table 1**.

### Sequence Read Mapping

A mean of 44.00 million raw read pairs per library were generated by sequencing of the pooled RNA samples. After filtering, a mean of 39.62 million read pairs (90.04%) remained, and a mean of 25.22 million (63.45%) were uniquely mapped to the ovine genome (Oar_rambouillet_v1). We observed a high number of reads that mapped to multiple genomic loci (mean of 34.39%) and further investigation indicated insufficient depletion of ribosomal RNAs (**Supplementary Figure 3**) (27). Consequently, only uniquely mapped reads were included in the analyses for the present study.

**Supplementary Figure 4** shows that the density of gene counts was similar in all libraries before (A) and after filtering (B) for lowly-expressed genes. The estimated biological coefficient of variation (BCV) is shown in **Supplementary Figure 5A**, while **Supplementary Figure 5B** shows the Quasi Likelihood dispersions. All RNA-seq analysis statistics are provided in **Supplementary Table 2**.

### Differential Expression Analysis

**Figure 2:**
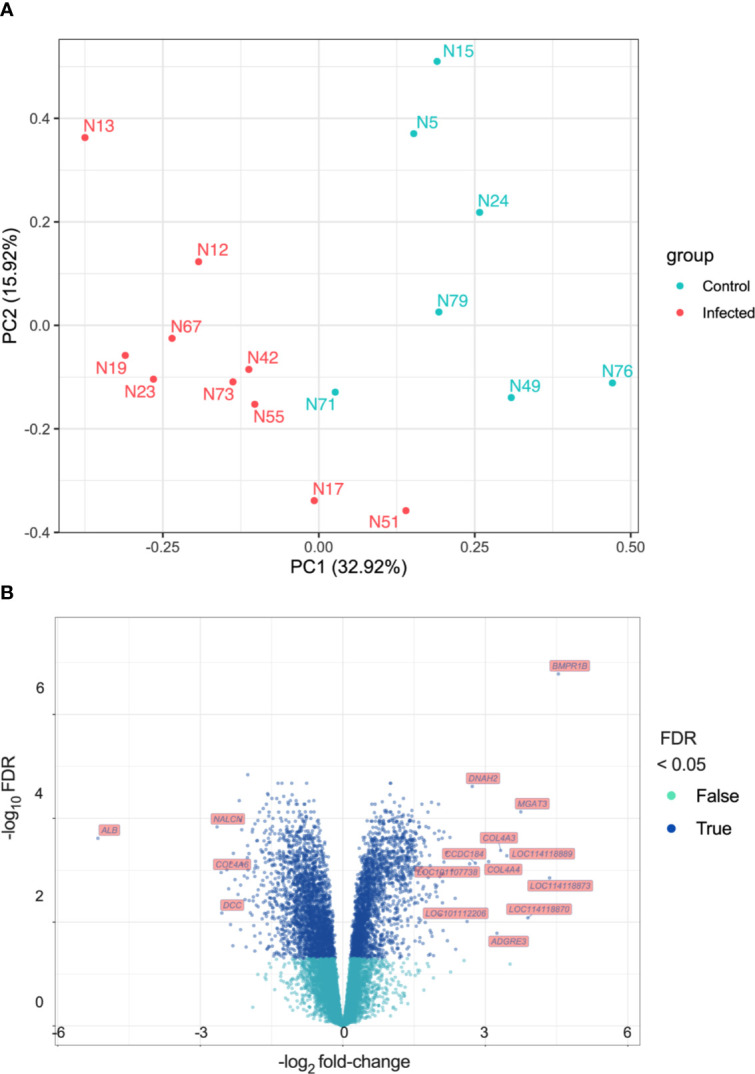
Differential Expression analysis **(A)** Principal Component Analysis (PCA). Principal components were calculated and the first two ranked by proportion of variation explained are shown (PC1 and PC2). **(B)** Volcano plot showing log_2_ fold-change (x-axis) and their −log_10_ FDR values (y-axis) for the DEGs detected.

The PCA plot (**Figure 2A**) indicates a clear separation of control and infected groups. However, samples N58 and N66 appeared to be outliers (**Supplementary Figure 6**). Animal N58 from the control group weighed only 24.6 kg at the beginning of the trial, which was considered a low value for a sheep of six to nine-months old. Additionally, haemoglobin levels from N58 were very low at the end of the trial (**Figure 1D**, lowest Hb point), being more similar to those in the infected group. Animal N66 from the infected group lost 3.3 kg. To further explore gene expression profiles, we performed hierarchical cluster analysis with and without the outlier samples. Exclusion of outlier individuals improved the clustering of groups of samples (**Supplementary Figure 7**). Given the possibility of N58 being a younger animal or another unrecognised underlying condition affecting both animals, and considering that the aim of the study was to explore the main differences between control and infected groups, not the natural individual variation of the response to infection, we performed all downstream analyses without samples N58 and N66. **Figure 2A** shows the PCA plot after outlier removal, where infected and control groups are clearly separated, particularly with regards to PC1 (32.92% of the overall variation).

There were 5,132 differentially expressed genes (DEGs) detected, 2,454 exhibiting increased expression and 2,678 exhibiting decreased expression. The log_2_ fold-change (log_2_FC) values for the DEGs varied between -5.16 (FDR = 2.42 × 10^-4^) for the albumin gene (*ALB*) to 4.54 (FDR = 1.66 × 10^-7^) for the bone morphogenetic protein receptor type 1B gene (*BMPR1B*). All DEGs and their statistical significance are shown in **Supplementary Table 2**. The volcano plot in **Figure 2B** represents all DEGs and shows labels for genes that meet the parameters: |log_2_ fold-change| > 2.5 and FDR < 0.05. **Table 1** shows DEGs that are of interest in the context of the immune response to *F. hepatica* infection.

**Table 1:** Selection of DEGs that are related to the immune response at the hepatic lymph node in chronic ovine infection with *F. hepatica*.

Gene symbol	log_2_ fold-change	FDR
***CD83***	0.703	9.48 × 10^-5^
***BCL6***	0.982	2.33 × 10^-4^
***TGFBR2***	-0.638	2.49 × 10^-4^
***IL12RB2***	-1.221	2.63 × 10^-4^
***IRF4***	0.628	6.49 × 10^-4^
***TLR4***	-0.769	6.51 × 10^-4^
***CASP7***	0.874	9.14 × 10^-4^
***SMAD2***	0.404	1 × 10^-3^
***STAT3***	-0.414	2 × 10^-3^
***CD40***	0.403	3 × 10^-3^
***FCER1G***	-1.009	3 × 10^-3^
***TLR5***	-1.281	5 × 10^-3^
***RORA***	-0.620	9 × 10^-3^
***CASP3***	-0.388	1.20 × 10^-2^
***SMAD4***	-0.219	1.40 × 10^-2^
***IL12B***	-1.357	1.58 × 10^-2^
***TGFB1***	-0.281	2 × 10^-2^
***TAB1***	-0.240	2.50 × 10^-2^
***ARG2***	0.424	3.70 × 10^-2^

### Ingenuity Pathway Analysis (IPA)

**Figure 3:**
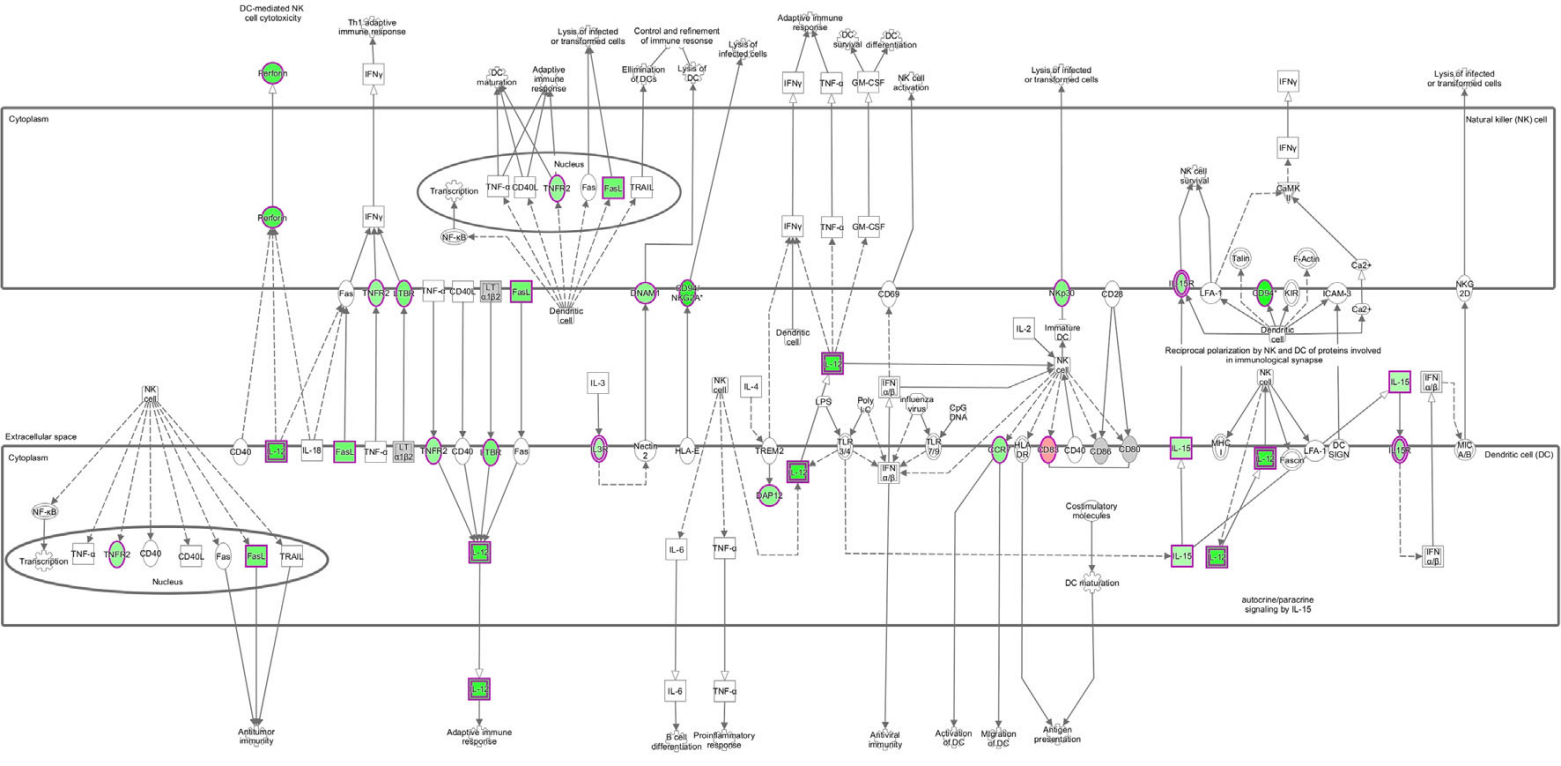
Crosstalk between DC and NK. Pathway represented with gene expression (log_2_ fold-change) values overlaid. Red shading indicates increased and green shading indicates decreased, while colour intensity indicates expression level. White and grey shading indicate not significantly differentially expressed and filtered out due to low expression, respectively. Solid and dashed lines represent direct and indirect interactions, respectively, between molecules. Open arrow means ‘translocate to’, solid arrow means ‘acts on’. Shapes indicate functional class of a gene product; vertical ellipse (transmembrane receptor), horizontal ellipse (transcription regulator), square (cytokines), circle (others), cogwheel (pathways). Double edge indicates complex or group. © 2000–2020 QIAGEN. All rights reserved.

The top ten enriched IPA canonical pathways are shown in **Table 2**. Many of these are related to DNA repair, cell cycle and metabolism. Other relevant pathways are also shown. **Figure 3** shows how the crosstalk between dendritic cells (DC) and natural killer (NK) cells was affected.

**Table 2:** Top 10 canonical pathways and other significant immune response related pathways.

Top 10 Pathways	Predicted activation	-log_10_ (*P*-value)	*Z*-score
Nucleotide excision repair pathway	Activated	7.41	2.524
Cell cycle control of chromosomal replication	Activated	5.37	3.742
Oxidative phosphorylation	Activated	4.05	4.243
Primary immunodeficiency signalling	No activity available	3.8	–
Crosstalk between DC and NK	Inhibited	3.57	-3.742
Role of BRCA1 in DNA damage response	Activated	3.55	3.317
Base excision repair pathway	No activity available	3.35	–
Mitotic roles of polo-like kinase	Activated	3.27	1.667
Nucleotic excision repair pathway	No activity available	3.02	–
Systemic lupus erythematosus signaling	No activity available	2.83	–
**Other relevant pathways**	**Predicted activation**	**-log_10_(*P*-value)**	***Z*-score**
T Helper cell differentiation	No activity available	2.37	–
Th1 and Th2 activation pathway	No activity available	2.12	–
Th1 pathway	Inhibited	1.84	-2.714
Cytotoxic T lymphocyte -mediated apoptosis of target cells	Inhibited	1.82	-2.236
STAT3 pathway	Inhibited	1.78	-2.828
B cell development	No activity available	1.71	–

Highly significant upstream regulators were mostly transcription factors functionally related to cell cycle, development or survival. However, among the predicted upstream regulators were molecules of particular interest, some of which have previously been shown to be relevant to *F. hepatica* infection. These are shown in **Table 3** in conjunction with relevant predicted master regulators resulting from the causal network analysis.

**Table 3:** Selection of significant upstream and master regulators.

Upstream regulator	Predicted activation state	*Z*-score	*P*-value of overlap
PTGER2	Activated	3.273	2.53 × 10^-7^
IL-4	Activated	2.670	2.96 × 10^-5^
IL-12	Inhibited	-2.882	1.27 × 10^-3^
IFN alpha/beta	–	-0.451	1.16 × 10^-2^
TCR complex	–	1.112	1.80 × 10^-2^
TNF receptor group	–	-0.254	4.48 × 10^-2^
**Master regulators**			
TLR1-TLR2 complex	Inhibited	-2.165	4.55 × 10^-6^
TH2 Cytokine group	Activated	3.046	1.21 × 10^-5^
GATA3	Activated	2.036	1.57 × 10^-5^
TLR3/4 group	–	-1.640	1.01 × 10^-4^
NFKB1	Inhibited	-3.159	2.18 × 10^-4^

From the 31 significant Toxicologic Functions encountered, 21 (67.7%) were related to liver damage or disease, while the ‘Parasitic Infection’ annotation was predicted to be activated (*P*-value = 4.27 × 10^-5^, *Z*-score = 2.35) in the Diseases and Functions output. Diseases and Functions also highlighted immune response relevant annotations that were all predicted to be decreased. These accounted for 50% of the overall decreased annotated output. Those predicted to be increased were related to cell cycle, cell death and survival, cellular function maintenance, infectious diseases, cancer or hepatic diseases. Complete tables for Canonical Pathways, Toxicologic Functions, Diseases and Functions and Upstream Analysis from IPA are provided in **Supplementary Table 3**.

### Gene Ontology and KEGG Pathways

Mapping of our data to one-to-one sheep to human orthologs (as ovine genes IDs cannot be used in IPA), resulted in the number of input DEGs being reduced to 1,402. We therefore performed further analyses using DAVID, which uses ovine gene IDs to retrieve gene information that is then used to query GO and KEGG, in order to obtain more information from our data set. The analysis of GO terms and KEGG pathways through DAVID does not allow for such an in-depth analysis as the one available in IPA. However, it has the benefit that the input data is the complete list of significant DEGs, offering a broader view of the processes involved.

**Figure 4:**
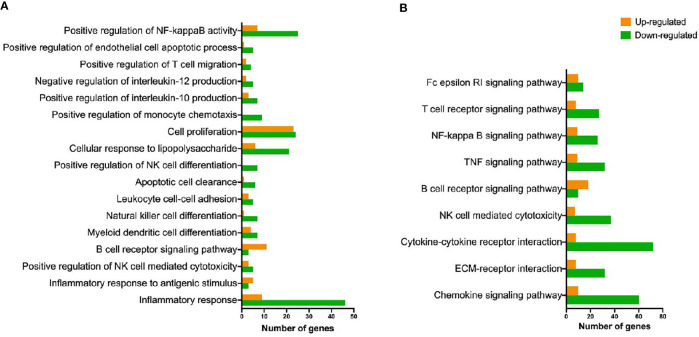
Representation of GO biological processes and KEGG pathways detected as up- and down-regulated. **(A)** Biological processes terms (gene ontology) overrepresented in ovine hepatic lymph node after *F hepatica* infection. **(B)** Overrepresented KEGG pathways found in ovine hepatic lymph nodes after *F hepatica* infection.

There were 104 significant terms identified by Gene Ontology analysis. **Figure 4A** shows a selection of relevant terms. Significantly enriched KEGG pathways of interest are included in **Figure 4B**. Further information about GO and KEGG results can be found in **Supplementary Table 4**.

## Discussion

The pathophysiology of chronic ovine fasciolosis is characterized by anaemia, hypoalbuminemia, eosinophilia and elevated liver enzymes (5). We observed that the infected animals from our trial showed a significant increase in the percentage of peripheral eosinophils by 2 wpi, which had increased further by 7 wpi as compared with control animals, with this difference declining by week 16. At 16 wpi, haemoglobin levels were significantly reduced in infected animals, with packed cell volume (PCV) showing a similar trend, although not significantly different. These changes are consistent with the well-described changes during chronic ovine fasciolosis (5).

Total plasma protein was also decreased in infected animals at 16 wpi, although again the magnitude of this change was not significant. However, our transcriptomic results indicate that albumin was the most highly down-regulated gene, with a log_2_ fold-change of -5.15 (FDR = 2.42 × 10^-4^). Albumin is one of the main plasma proteins, and it is responsible for maintaining osmotic pressure, binding and transport of molecules and acting as an antioxidant during oxidative stress processes or as an immunomodulator (28, 29). It is mainly synthetized in the liver, and during the parenchymal stage of infection, liver damage produced by the migrating parasites leads to a decrease in albumin production (30). However, expression and/or production of albumin from other tissues such as the mammary gland or lymph nodes in cattle has also been documented (31). At more chronic stages, loss of blood may also contribute to the reduction of overall albumin plasma concentrations (5). A previous RNA-seq study using liver tissue from sheep infected with *F. hepatica* did not find downregulation of albumin transcripts, as we did in the hepatic lymph node (13). However, the authors clarify that for their study, liver tissue from macroscopic lesions that showed fibrosis and eosinophilia under histological observation was used. It is therefore likely that few hepatocytes were included and if any were present, albumin transcripts did not have relevance in the overall transcriptome. Our results indicate that reduced blood albumin levels during fasciolosis may not exclusively be due to liver tissue damage or blood loss, and that other mechanisms involving the hepatic lymph node and other tissues can also contribute to reduce overall albumin levels.

### Hyperplasia of the Lymph Node Is Not Dependent on Fibrosis or TGF-β Signalling

As mentioned above, the penetration, and migration of the parasites through the liver and bile ducts can damage the tissue, with consequences like fibrosis, subcapsular haemorrhage, and cholangitis. This is the result of mechanical disruption by the parasite’s oral and ventral suckers and body spines, the action of proteases released by the parasite and the healing process initiated by the host in response to these (5). It is therefore unsurprising that previous studies, both in cattle and sheep, observed up-regulation of the transforming growth factor pathway, as it is involved in the generation of fibrosis and collagen deposition, as well as mediation of immune tolerance and immune regulation (32, 33). In sheep, the *TGFB1* and *COL1A1* genes were upregulated during the acute phase of infection in peripheral blood mononuclear cells (PBMC), and at later stages in liver tissue (8 wpi) (7, 13). Expression of *TGFB1* in the liver tissue of chronically infected sheep has been shown to be significantly correlated with fluke burden and faecal egg counts (FEC) (34). Although we report here the collagen genes *COL4A3* and *COL4A4* to be among the most up-regulated genes in our data set, analysis of KEGG pathways demonstrated an overall downregulation of the extracellular matrix (ECM) receptor interaction. Furthermore, we observed reduced expression of several genes involved in the TGF-β signalling pathway, including *TGFB1*, *TGFBR2*, *TAB1* and *SMAD4*. Our study however shows data from later stages of infection (16 wpi). We report here a significant hyperplasia of lymph nodes from infected sheep as compared to uninfected animals, which is in agreement with previous observations (35). However, our results indicate that this enlargement was not related to fibrosis of the lymph node tissue or that an alternative mechanism for fibrosis was taking place. In fact, pathological examination of the lymph nodes from infected animals did not find signs of fibrosis in the tissue. It is likely that the infiltration by immune cells is more relevant for the increased size of the organ than fibrosis, while expression of TGF-β is localized in the liver and detectable in PBMC due to their “echoing” capacity, but not in the hepatic lymph node (6).

### Immune Response Against *F. hepatica* in the Hepatic Lymph Node Is Primarily T_h_2

Regarding the local immune response, our study reveals an inhibition of many pro-inflammatory processes shown by IPA and DAVID outputs. Some of these have previously been identified and are a hallmark of infection with *F. hepatica*. For example, the inhibition of the T_h_1 pathway shown in the enriched IPA canonical pathways, the downregulation of the ‘Inflammatory Response’ GO biological process and the ‘TNF signalling pathway’ in KEGG, and the activation state of the T_h_2 cytokine group in the IPA master regulators. The transcription factor IRF4, which was slightly up-regulated in infected animals, participates in the development of M2 macrophages and plasma cells, and initiates T_h_2 and T_h_17 cell differentiation through DCs (36–38). Another indicator of alternative activation of macrophages, arginase-II (*ARG2*), was similarly activated (39, 40). Additionally, master regulator *GATA3*, critical in T_h_2 cell differentiation was predicted to be activated (41).

TGF-β participates in the differentiation of Treg and T_h_17 cells (42, 43). The TGF-β pathway may be a mechanism employed by *F. hepatica* to drive the host immune response by releasing FhTLM, a protein that can engage with TGF-β receptors I and II and initiate signalling through the Smad2/3 molecule (44). Flynn et al. (45) reported that in PBMC isolated from *F. hepatica*-infected cattle, neutralisation of TGF-β *in vitro* resulted in an increased production of IL-4 (45). We did not find the TGF-β signalling pathway to be altered significantly in our data, which may be due to the nature of the tissue being analyzed or the longer course of our study compared to previous work. Furthermore, expression of the *STAT3* gene, also involved in T_h_17 and T follicular helper (Tfh) cell differentiation was reduced in infected sheep and the STAT3 canonical pathway was predicted to be inhibited in IPA (46). IL-21, which is mainly produced by CD4+ T cells and NKT cells, and is involved in the differentiation of Tfh and T_h_17, was up-regulated (47). However, we found the expression of master regulator of T_h_17 cells RORα (RORA), to be downregulated (48). Unlike the study by Alvarez Rojas et al. (13) where no particular tendency towards a T-cell type was encountered in liver tissue from infected sheep at 8 wpi, our data indicates that a predominant activation of T_h_2 cells takes place in the hepatic lymph node, with some indication of Tfh activity, but an inhibition of T_h_1 cells. It is possible that liver tissue and lymph node show different expression patterns, or that the longer length of our study (16 weeks) allowed for further T_h_2 cell activation.

As Tfh cells are mainly located in secondary lymphoid organs like lymph nodes, we aimed to explore further their role during infection. Tfh are a specialised subset of CD4+ T cells that are critical for B cell differentiation into plasma and memory cells in the germinal centres. They fulfil their function *via* CD40 interaction with B cells, and the production of IL-21 which drives their proliferation (49, 50). In our data set, both CD40 and IL21 were activated in infected animals. Additionally, Tfh are characterised by the expression of the transcription factor B cell lymphoma 6 gene (*BCL6*), which we found to be up-regulated (51). Previous work has shown that *F. hepatica* soluble products induce Tfh differentiation through DC *via* the C-lectin receptor specific signalling pathway (DC-SIGN) (52). On the other hand, although it was not possible to predict its activation status, B cell development was among the significantly enriched pathways in IPA, and both GO and KEGG pathway overrepresentation analyses show an increased representation of B cell signalling pathways with a larger number of up-regulated genes. Furthermore, we report an increased expression of immunoglobulin genes, which are among the 10 most highly expressed genes in our data set. These data show an overall activation of B cell activity. We therefore found an activation of T_h_2 pathways and B cell signalling, a pattern characteristic of immune responses to helminths that has been shown to induce protective immunity to intestinal nematodes like *Trichuris muris* in mice, for instance (53).

### IgE- Dependent Processes Are Inhibited

Another feature of T_h_2 responses is production of helminth-specific IgE antibodies. Mast cells and eosinophils then bind to the helminth’s surface *via* IgE Fc receptors and degranulate, releasing mediators that start a hypersensitivity reaction key for IgE-dependent killing (54, 55). A comparison of eosinophil and IgE kinetics during *F. gigantica* infection in Merino sheep and the resistant Indonesian Thin tail (ITT) breed showed that the latter have higher eosinophil levels throughout infection and an enhanced IgG1, IgM and IgE response early post-infection as compared to the non-resistant animals (56). Our results show a decreased expression in infected animals of Fc fragment of IgE receptor Ig (*FCER1G*), the high affinity IgE receptor. This receptor is expressed in mast cells, basophils, eosinophils, and DC among others, and participates in IgE-mediated antigen presentation (57, 58). FcϵRI has been shown to be involved in effective eosinophil-mediated cytotoxicity against *Schistosoma mansoni* (59). KEGG pathway overrepresentation analysis from our data also identified the FcϵRI signalling pathway to be significantly enriched, showing more genes down- than up-regulated genes. Histological examination of *F. hepatica* infected sheep liver has previously demonstrated infiltration with eosinophils in varying numbers (35). Mast cell numbers in the liver and peritoneal fluid of mice after *F. hepatica* infection increased, and although there is little evidence that this happens in sheep, infiltration of mast cells in the liver of infected cattle have also been reported (60–62). Whether mast cells and IgE-mediated processes play a key role in the response to *F. hepatica* infection still needs to be elucidated, but there is evidence that *F. hepatica* tegumental antigens can impair mast cell activity (63).

### Natural Killer Cell Activity Is Reduced

We found that in hepatic lymph nodes from infected sheep there is a consistent transcriptional perturbation of processes involving Natural Killer (NK) cells, such as inhibition of differentiation, regulation and cytotoxicity. NK cells are granular lymphocytes which are important in innate immune responses. They are involved in orchestrating cell-mediated cytotoxicity through their direct recognition of pathogen-associated molecular patterns (PAMPs) through receptors such as Toll-like receptors (TLRs) including TLR2, TLR3, TLR5 and TLR9, among others (64–66). Along with the inhibition of the various NK cell pathways, we observed a predicted inhibition of the TLR1-TLR2 complex, a negative *Z*-score for the TLR3/4 group (although no prediction was made because Z-score was higher than -2), and a reduction in expression of the *TLR5* gene (log_2_ fold-change = -1.28, FDR = 5.86 × 10^-3^)

The importance of NK cell activity in orchestrating protective immunity against other parasitic helminths has previously been documented. For instance, protective vaccination of cattle against the parasitic nematode *Ostertagia ostertagi*, and posterior *in vitro* re-stimulation of abomasal lymph node cells produced a dominant proliferation of NK cells (and not B cells, αβ-T cells or γδ-T cells) (67). During an infection trial with *Cooperia oncophora* in calves, NK cell proliferation increased (along with proliferation of T and B cells) after *in vitro* re-stimulation of mononuclear cells from mesenteric lymph nodes (68). More recent work on *Heligmosomoides polygyrus bakeri*, a natural parasitic roundworm of mice, has shown that recruitment of cytotoxic NK cells to the intestine early in infection increases host tolerance by mediating vascular integrity and endothelial cell survival (69). Very little published information regarding the role of NK cells during fasciolosis is available. Jedlina et al. (70) found a significantly increased frequency of cytotoxic NK cells (higher than CD4+ or CD8+ T cells) in the peritoneal fluid of rats infected with *F. hepatica* by 4 days post-infection (dpi). This declined at 7 dpi, which the authors attributed to a change from cytotoxic to regulatory functions (70). Another study, also in rats, showed an increased percentage of IFN-γ producing NK cells in the liver tissue at 7 dpi, which remained elevated until day 28 (71). Rojas et al. (12) reported a down-regulation of NK cell mediated cytotoxicity in sheep at 2 wpi, but not at later stages in PBMCs (12). It is possible that during early infection the impact on NK cell activity is even greater, and also detectable in PBMCs. Overall, our results show that the activity of NK cells during chronic fasciolosis in the hepatic lymph node of sheep is decreased, which adds to our knowledge of the immunoregulatory effects of *F. hepatica*.

### Dendritic Cells and Their Interactions Are Affected

NK cells also participate in the adaptive immune response by interacting with DC to drive adaptive responses, and prime T_h_1 cells *via* IFN-γ and TNF production (72, 73). We show here that the crosstalk between DC and NK pathways is inhibited, indicating that an array of both innate and adaptive immunological processes are affected by the immunomodulatory actions of *F. hepatica*. DC are an important source of IL-12, which is a proinflammatory cytokine that promotes both NK cell cytolytic activity and the differentiation of T_h_1 cells (74). Production of IL-12 is therefore critical for developing an effective adaptive immune response, as its impairment can block DC-induced IFN-γ responses (75). Fu et al. (7) found that both *IL12* and *IL18* were downregulated in PBMC from sheep with fasciolosis, and hypothesized that the IgG isotype bias of immunoglobulins towards IgG2 generally observed in infected animals was a result of the attenuated effect of both cytokines on B cells (7). Our results show the expression of *IL12B* to be significantly reduced in infected animals, and the IL-12 complex predicted to be inhibited. However, *IL18* was not among the DE genes nor the statistically significant pathways/upstream regulators. This may indicate a more important role of IL18 in the peripheral response. One molecule that may be partly responsible for the reduced *IL12* expression detected in our study may be prostaglandin E_2_ (PGE2). We found PGE2 receptor 2 (*PTGER2*) within the upstream regulators predicted to be activated. PGE2 has been shown to have strong immune suppressor functions, known to be increased during alternative activation of macrophages and participating in the suppression of IL-12 synthesis (76). As a parasitic example, *Schistosoma mansoni* can induce PGE2 production by human and mice keratinocytes, which in turn stimulates IL-10 production, key for survival of the parasite in the skin (77). *In vitro* experiments have demonstrated that molecules released by *F. hepatica* induce PGE2 and IL-10 secretion by murine macrophages (78, 79).

The activity of DC has previously been shown to be affected by *F. hepatica* products in numerous studies, with reduced IL-12 production being one of the consequences. For instance, *F. hepatica* antigens affect DC maturation and function by supressing cytokine production (including IL-12p70, IL-6, TNF, and nitrite) and cell surface marker expression (CD80, CD86, and CD40) from murine DCs *in vitro* (80, 81). KEGG pathways and GO terms showed that the NF-κB transcription factor signalling pathway, which is related to the regulation of gene expression associated with pro-inflammatory processes including DC activation, was also downregulated (82, 83). It has previously been established that *F. hepatica* tegumental antigens suppress LPS-induced NF-κBp65 production by DCs (84). Furthermore, Falcón et al. (80) found that DC stimulated with *F. hepatica* excretory-secretory products (ESP) primed CD4+ cells towards a T_h_2/regulatory response, with increased production of IL-4, IL-5, IL-10 and TGF-β, and reduced IFN-γ. Some of these changes have been observed in sheep, where infection with *F. hepatica* produced hyperplasia of the hepatic lymph nodes with increased numbers of DC at 18 dpi, but a significant decrease in the expression of their antigen presentation markers (CD83 and MHC-II) (85). Our results indicate an increased expression of both the *CD83* and *CD40* genes, which could indicate a difference between acute and chronic infections. CD40 does not appear to be activated in **Figure 3** this sheep gene did not have a one-to-one human ortholog and so was excluded from IPA analysis.

### Apoptosis Is Not Induced in the Lymph Node

*Fasciola hepatica* infection can induce apoptosis of peritoneal leucocytes and PBMC of sheep during acute disease and of liver eosinophils during chronic disease (7, 86, 87). However, our data reveals that the *PRF1* gene (encoding perforin1) has a decreased expression in infected animals compared to controls. Perforin is one of the main molecules involved in DC–NK communication, and also in the targeted apoptosis by NK cells and cytotoxic T lymphocytes, playing an important role in killing other cells that are recognized as non-self by the immune system (88). Our results indicate that the canonical pathway ‘Cytotoxic T lymphocyte -mediated apoptosis of Target cells’ was also predicted to be inhibited. Caspases are molecules also involved in the apoptotic process (89). While caspase 7 is shown to be activated in our data set, caspase 3 has been shown to be the primary trigger of apoptosis, with cell death being more efficient in the presence of this molecule (90). We found *CASP3* expression to be downregulated, and our data therefore shows that in our trial infection, apoptosis was not induced in the hepatic lymph node during infection. Finally, apoptosis can be triggered by TNF- induces signaling. Therefore, downregulation of ‘TNF-signaling pathway’ may also be involved in the reduced apoptosis observed (91).

### Concluding Remarks

Various aspects of the host response to infection with *F. hepatica* both in cattle and sheep have been explored to date. However, this is the first study where the transcriptomic response in the hepatic lymph node of sheep has been characterised. Some of our findings confirm those changes previously identified as being characteristic of infection with *F. hepatica*. These included the downregulation of T_h_1 cell types, up-regulation of T_h_2 or B cell signalling, and an impact on DC functions. Other pathways that have previously been shown to be activated did not show this pattern in our study, for instance TGF-β-mediated fibrosis or apoptosis. The reasons for this may include tissue, host, or methodological variations. We provide stronger evidence of inhibition by *F. hepatica* infection of NK cells and new evidence of its impact on IgE-dependent activity, both of which are involved in the release of cytotoxic molecules. We propose that inhibiting the activity of NK cells and of IgE may be a mechanism employed by *F. hepatica* to evade cytotoxicity. These results point to the possibility of targeting innate immunity actors signalling in future vaccination attempts. Given the considerable challenges in developing vaccines against this parasite, it is clear prototypes capable of counteracting the multiple immunomodulatory strategies employed by the parasite will be required. In this respect, experiments involving adjuvants that target NK cell activation may be useful.

## Data Availability Statement

The datasets presented in this study can be found in online repositories. The names of the repository/repositories and accession number(s) can be found below: https://www.ebi.ac.uk/ena, PRJEB44063.

## Ethics Statement

This experiment was approved by the Bioethics Committee of the University of Córdoba (UCO, Spain) (code No. 1118) and conducted in accordance with European (2010/63/UE) and Spanish (RD 1201/2005) Directives on animal experimentation. Written informed consent was obtained from the owners for the participation of their animals in this study.

## Author Contributions

VM-H, AM-M, and JP conceived and designed the experimental trial. VM-H collected samples from animals and carried out tissue isolation. AN-L performed laboratory work and analyzed data. JB supported lab work and provided technical advice and input on RNA isolation and library preparation. CC and DM participated in data analyses and bioinformatics. AN-L and GM contributed to the writing of the manuscript. VM-H, JP, GM, CC, and DM contributed to the editing of the manuscript critically for important intellectual content. All authors contributed to the article and approved the submitted version.

## Funding

This work was supported by the a Science Foundation Ireland grant (14/IA/2304) and a European Union Horizon 2020 programme (PARAGONE: vaccines for animal parasites. H2020-EU.3.2. SOCIETAL CHALLENGES, under grant agreement No 635408).

## Conflict of Interest

The authors declare that the research was conducted in the absence of any commercial or financial relationships that could be construed as a potential conflict of interest.

## Supplementary Material

Supplementary Figure 1 Lymphocytes, Monocytes, Neutrophils, Basophils and PCV at **(A)** 2, **(B)** 7 **(C)** and 16 weeks post infection in infected and uninfected animals.

Supplementary Figure 2 Histopathology of the hepatic lymph nodes. **(A)** Node from an uninfected control sheep showing a narrow cortex (c) with a few lymphoid follicles showing small germinal centers (arrows) and a large medulla (m). H&E staining, x20. **(B)** Node from an infected sheep showing large cortex composed of numerous lymphoid follicles with large germinal centers (arrows), H&E staining, x20. **(C)** Node from an uninfected sheep showing medulla (m) with indistinct medullary cords, H&E staining, x200. **(D)** Node from an infected sheep showing medulla with large medullary cords (arrows) due to increased number of lymphocytes, H&E staining, x200. **(E)** Node from an infected sheep showing medulla detail with infiltration of eosinophils (arrows), H&E staining, x400.

Supplementary Figure 3 Example of visualization of four samples using Integrative Genomic Viewer (IGV) showing reads mapping rRNA genes.

Supplementary Figure 4 Density of **(A)** raw **(B)** and filtered gene counts per sample.

Supplementary Figure 5 Dispersion parameters. **(A)** Scatterplot of the biological coefficient of variation (BCV) against the average log_2_ CPM of each gene. **(B)** Plot of the quarter-root QL dispersion against average log_2_ CPM of each gene.

Supplementary Figure 6 Principal Component Analysis (PCA) showing samples N58 and N66 that were removed from the analysis.

Supplementary Figure 7 Hierarchical Cluster dendrograms. **(A)** Clusters including samples N58 and N66. **(B)** Clusters excluding samples N58 and N66.
