# Supplementary figures and images for: Transcriptomic Analysis of Ovine Hepatic Lymph Node Following Fasciola hepatica Infection – Inhibition of NK Cell and IgE-Mediated Signaling
Source: Front Immunol. 2021 May 28;12:687579. doi: 10.3389/fimmu.2021.687579 (PMC8194261; doi:10.3389/fimmu.2021.687579)

**A****2 wpi**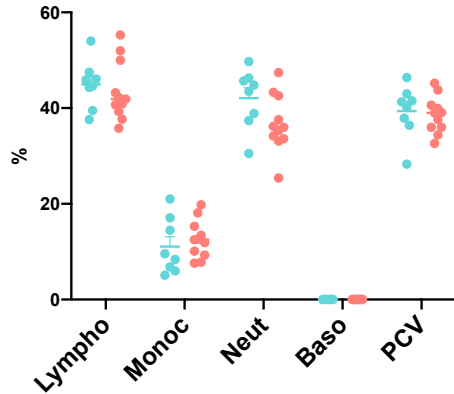**B****7 wpi**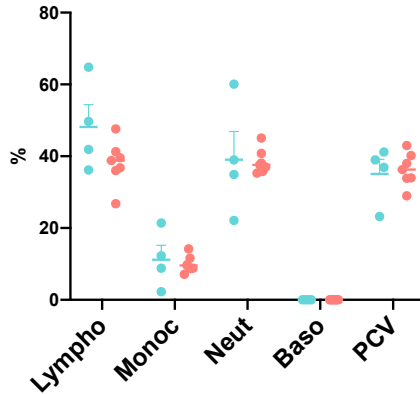**C****16 wpi**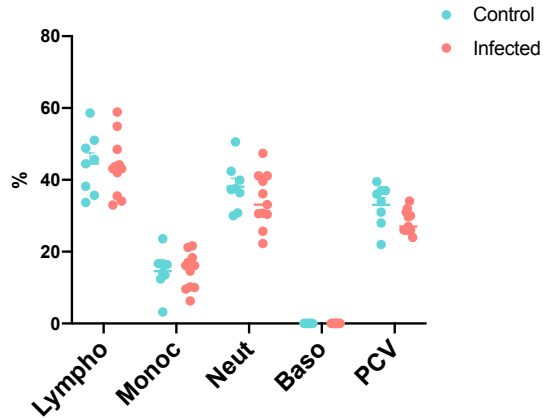

Supplement: Supplementary Figure 1 — Lymphocytes, Monocytes, Neutrophils, Basophils and PCV at (A) 2, (B) 7 (C) and 16 weeks post infection in infected and uninfected animals. [file Image_1.pdf]

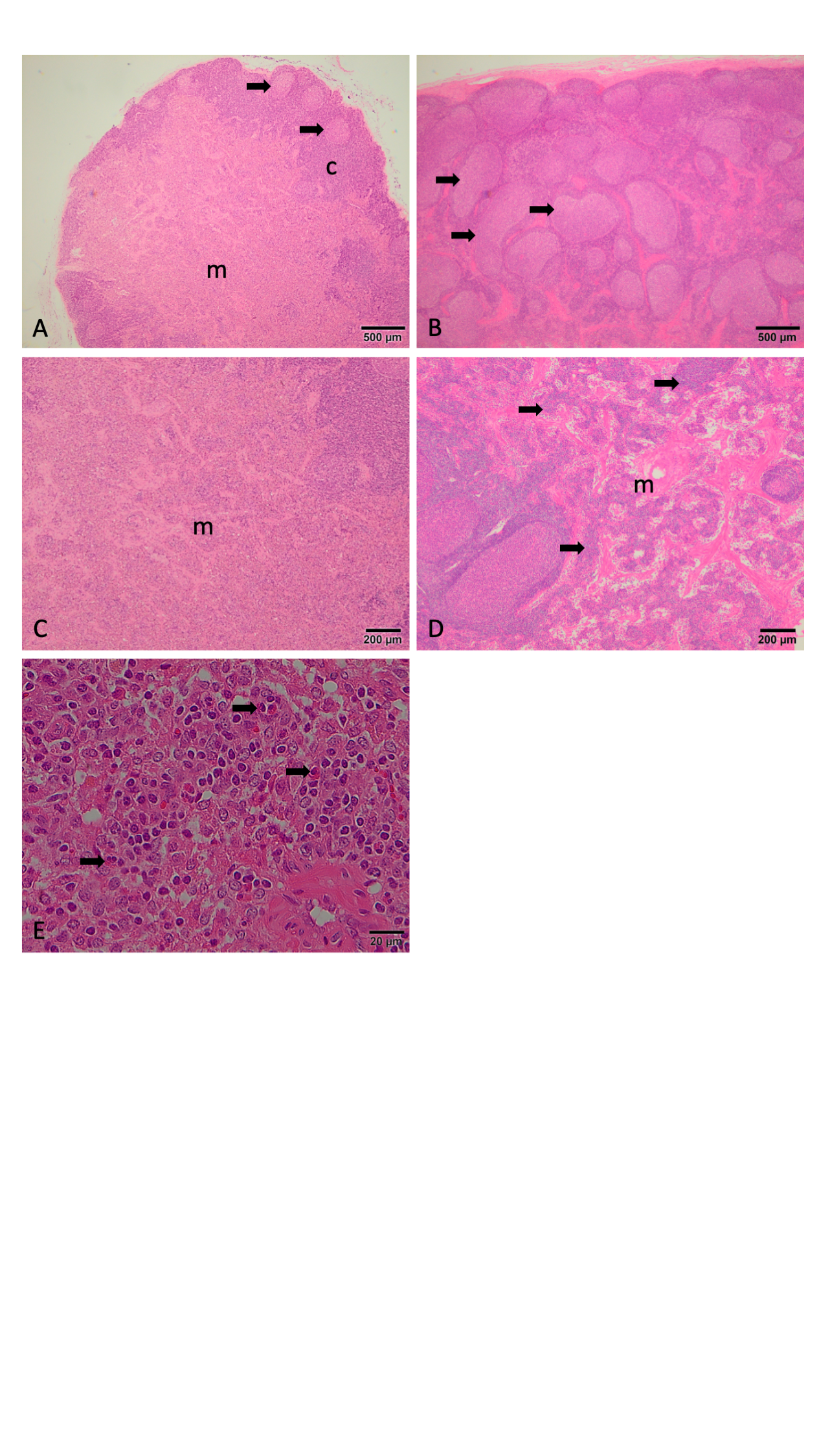

Supplement: Supplementary Figure 2 — Histopathology of the hepatic lymph nodes. (A) Node from an uninfected control sheep showing a narrow cortex (c) with a few lymphoid follicles showing small germinal centers (arrows) and a large medulla (m). H&E staining, x20. (B) Node from an infected sheep showing large cortex composed of numerous lymphoid follicles with large germinal centers (arrows), H&E staining, x20. (C) Node from an uninfected sheep showing medulla (m) with indistinct medullary cords, H&E staining, x200. (D) Node from an infected sheep showing medulla with large medullary cords (arrows) due to increased number of lymphocytes, H&E staining, x200. (E) Node from an infected sheep showing medulla detail with infiltration of eosinophils (arrows), H&E staining, x400. [file Image_2.tiff]

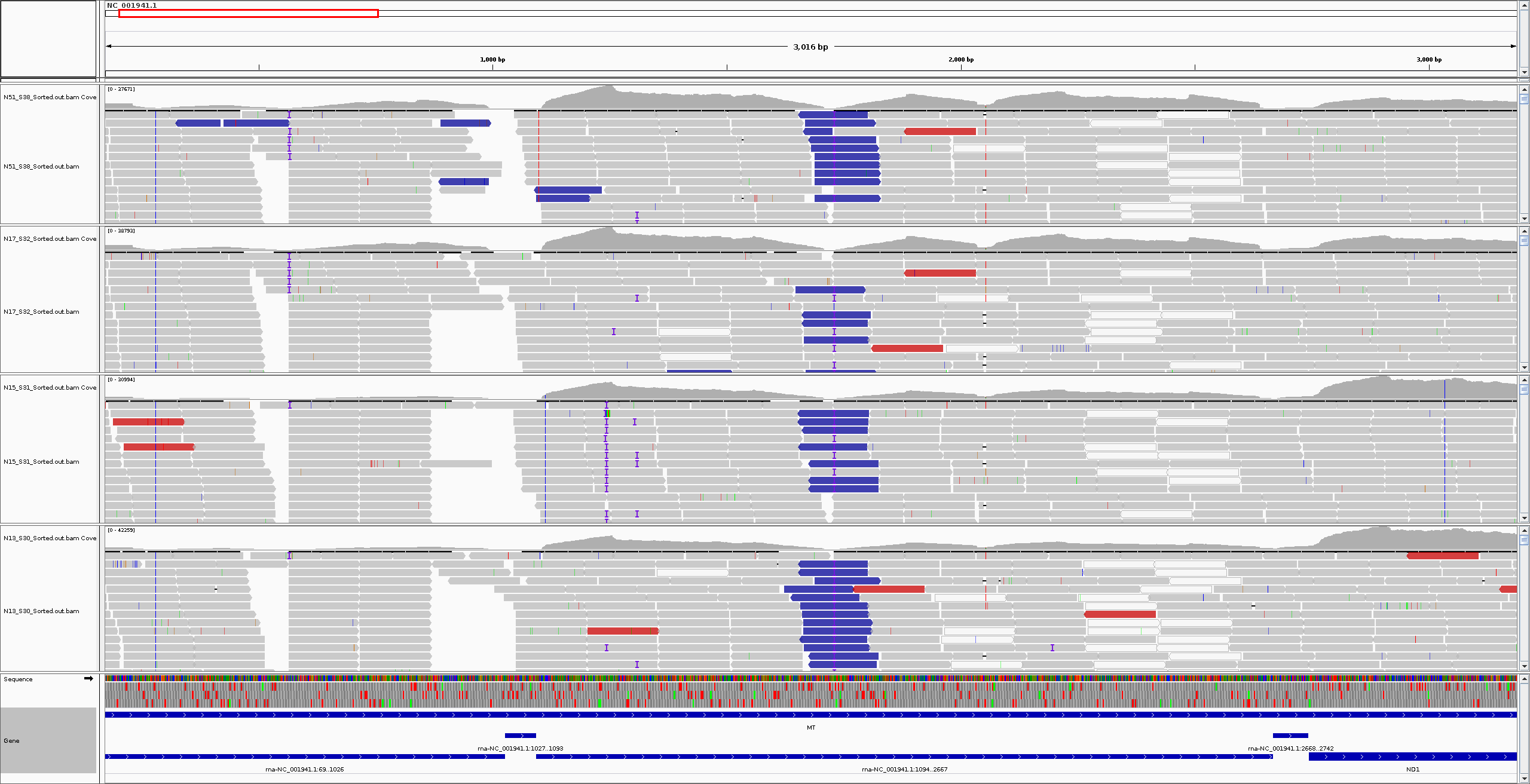

Supplement: Supplementary Figure 3 — Example of visualization of four samples using Integrative Genomic Viewer (IGV) showing reads mapping rRNA genes. [file Image_3.png]

**A**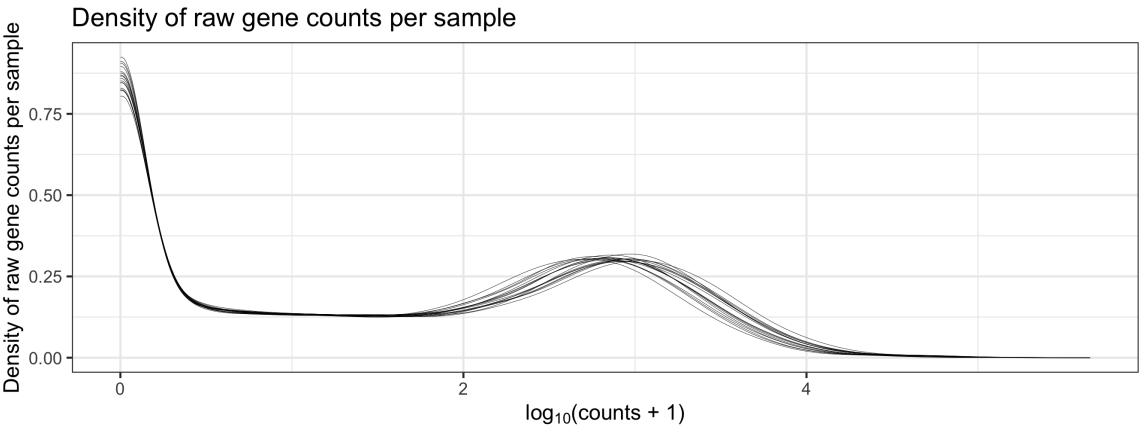**B**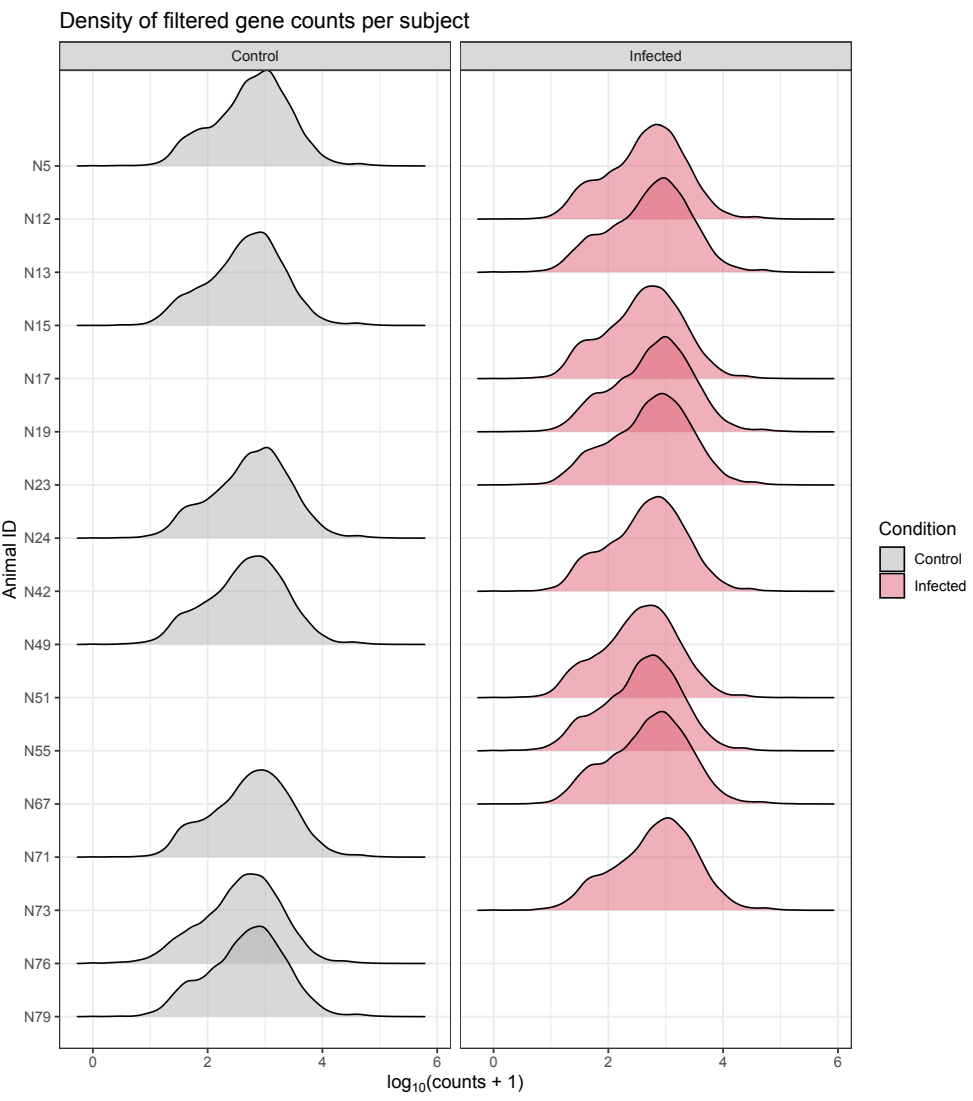

Supplement: Supplementary Figure 4 — Density of (A) raw (B) and filtered gene counts per sample. [file Image_4.pdf]

A

Estimated dispersions (NB model)

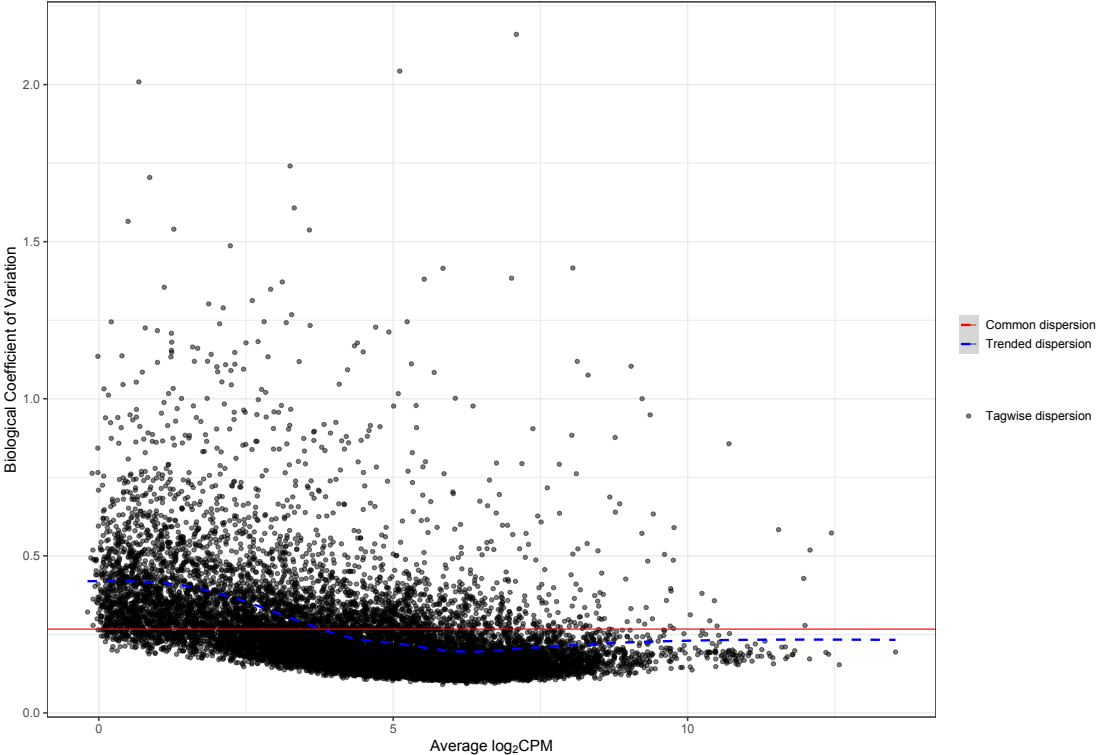

B

Estimated QL dispersions

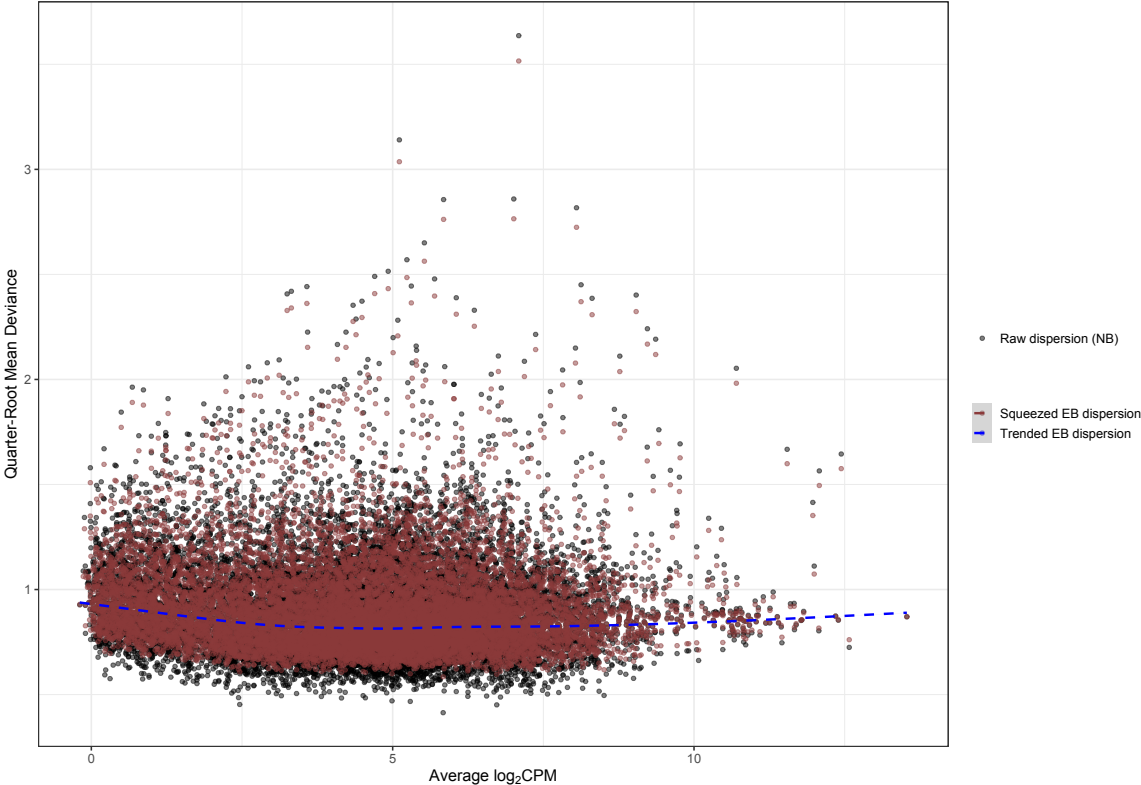

Supplement: Supplementary Figure 5 — Dispersion parameters. (A) Scatterplot of the biological coefficient of variation (BCV) against the average log2 CPM of each gene. (B) Plot of the quarter-root QL dispersion against average log2 CPM of each gene. [file Image_5.pdf]

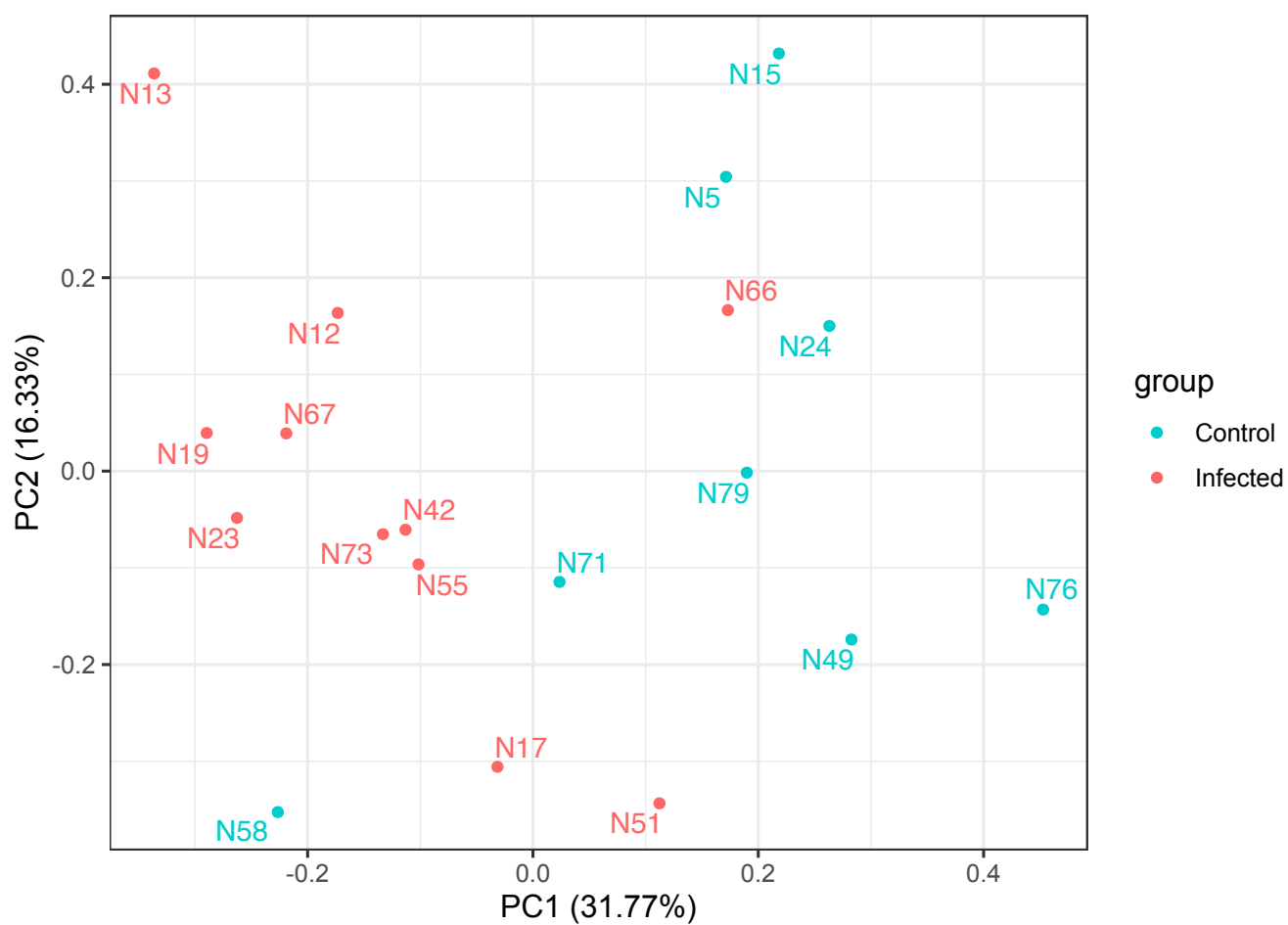

Supplement: Supplementary Figure 6 — Principal Component Analysis (PCA) showing samples N58 and N66 that were removed from the analysis. [file Image_6.pdf]

**A****Cluster dendrogram showing all samples**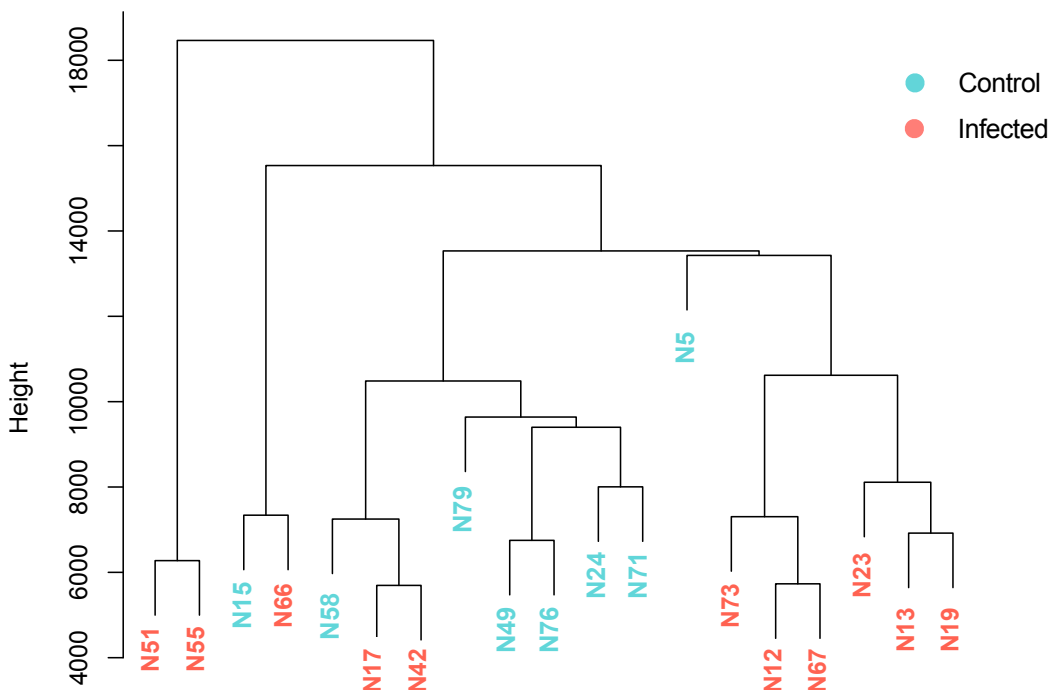**B****Cluster dendrogram without N58 and N66**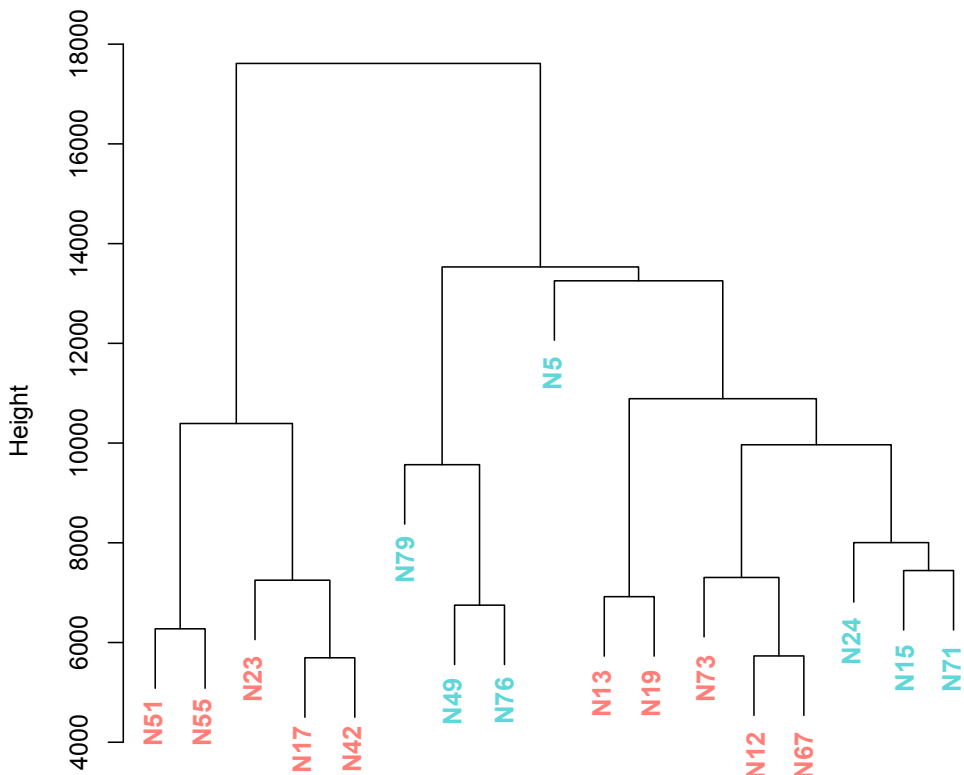

Supplement: Supplementary Figure 7 — Hierarchical Cluster dendrograms. (A) Clusters including samples N58 and N66. (B) Clusters excluding samples N58 and N66. [file Image_7.pdf]
